# Complete human CD1a deficiency on Langerhans cells due to a rare point mutation in the coding sequence

**DOI:** 10.1016/j.jaci.2016.05.028

**Published:** 2016-12

**Authors:** Daniela Cerny, Duyen Huynh Thi Le, Trung Dinh The, Roland Zuest, Srinivasan KG, Sumathy Velumani, Chiea Chuen Khor, Lucia Mori, Cameron P. Simmons, Michael Poidinger, Francesca Zolezzi, Florent Ginhoux, Muzlifah Haniffa, Bridget Wills, Katja Fink

**Affiliations:** a Singapore Immunology Network, A*STAR, Singapore; b School of Biological Sciences, Nanyang Technological University, Singapore; c Oxford University Clinical Research Unit, Hospital for Tropical Diseases, Ho Chi Minh City, Vietnam; d Genome Institute of Singapore, A*STAR, Singapore; e Experimental Immunology, Department of Biomedicine, University Hospital Basel, University of Basel, Basel, Switzerland; f Nuffield Department of Clinical Medicine, Oxford University, Oxford, United Kingdom; g Department of Microbiology and Immunology, University of Melbourne, the Peter Doherty Institute of Infection and Immunity, Carlton, Victoria, Australia; h Institute of Cellular Medicine, Newcastle University, Newcastle, United Kingdom

To the Editor:

The family of CD1 molecules is structurally similar to MHC class I molecules, but the 2 protein families mediate fundamentally different immune functions. MHC class I molecules present peptides to T cells, whereas CD1 molecules present lipids to natural killer T cells and other CD1-restricted T cells.^1^ CD1a is highly expressed on human Langerhans cells (LCs), a specialized mononuclear phagocyte that is prevalent in the epithelial cell layer of the skin and mucosal surfaces. Epidermal LCs can function as classical antigen-presenting cells (APCs) to induce naive T-cell responses in draining lymph nodes, but also have a regulatory function in the skin via local induction of regulatory T cells and maintenance of epithelial barrier integrity.^2^, ^3^ Human dermal dendritic cells (DCs) also express CD1a, but in much lower amounts compared with LCs. CD1a^+^ dermal DCs, which coexpress CD1c, have been shown to efficiently stimulate CD4^+^ and CD8^+^ T cells *in vitro*.^4^, ^5^ However, immune deficiencies due to selective CD1a defects have not been previously described, and it has proved difficult to dissect the specific role of CD1a in immune regulation.

During the course of a clinical study that involved analysis of APC subsets in human skin biopsies by flow cytometry, we identified a healthy Vietnamese individual, donor 007, who showed complete absence of CD1a expression on skin APCs (Fig 1, *A*). This case presented an opportunity to study the biological significance of CD1a expression. To check whether LCs were absent altogether in donor 007, we obtained a second skin biopsy, separated the epidermis from the underlying structures, and stained the epidermal tissues with antibodies binding to CD1a and to HLA-DR. Donor 007 LCs displayed intense HLA-DR staining with typical dendritic morphology, but CD1a staining was minimal (Fig 1, *B*).

We next addressed whether the CD1a deficiency represented a generic expression defect, using monocyte-derived dendritic cells (moDCs) as a model. In keeping with our earlier observations, moDCs from donor 007 showed no surface CD1a expression by flow cytometry or immunohistochemistry (see Fig E1, *A*, in this article's Online Repository at www.jacionline.org), in contrast to moDCs derived from a normal healthy control donor. Staining with other anti-human CD1a clones, OKT6 and NA1/34-HLK, showed the same result as staining with clone HI149 (see Figs E2 and E3 in this article's Online Repository at www.jacionline.org). In addition, no costain with early endosome antigen-1 and CD1a was observed, excluding CD1a accumulation in early endosomes in donor 007 (Fig E1, *B*).

To address whether the CD1a defect was caused by a mutation in the CD1a gene, we invited the parents and all 4 siblings of donor 007 for a clinical assessment and CD1a expression analysis. Summary clinical information for the family members is presented in Table E1 in this article's Online Repository at www.jacionline.org. Apart from donor 007's father, who had severe Parkinson's disease, the family members were generally healthy and displayed apparently normal skin barrier function and wound healing.

Both parents (001 and 002) and siblings 003, 004, and 006 showed normal CD1a surface expression on skin DCs and/or moDCs by immunohistochemistry and flow cytometry (Fig E4, *A*, in this article's Online Repository at www.jacionline.org). However, skin DCs of sibling 005 showed complete absence of surface CD1a expression, similar to donor 007 (Fig E4, *B*). Blood DC subsets from family members, and from Singaporean healthy controls, were also analyzed by flow cytometry; the absence of CD1a had no impact on the development of blood DC subsets, and did not affect the expression of CD1c and CD1d molecules, excluding an intracellular CD1 protein trafficking defect (Fig E4, *C*-*F*).

To establish the genetic cause of the CD1a deficiency, we isolated RNA from moDCs for CD1a mRNA length and sequence analysis (see Fig E5, *A*, in this article's Online Repository at www.jacionline.org). The lengths of the CD1a open reading frame from donor 007, from the parents, and from 1 sibling were identical, ruling out a shorter splice variant as the cause of the CD1a expression defect in donor 007. However, sequencing of the mRNA identified a single nucleotide polymorphism (SNP) (rs761269454) (Fig E5, *B*) that differed between donor 007 and nonaffected family members. The rs761269454 T to C conversion results in an amino acid change from Leucine to Proline at position 285 of the CD1a protein, located in the α3 domain of CD1a (Fig 2, *A*). Interestingly, parent 001 exhibited a double peak at this nucleotide position, suggesting that both the normal and mutant allele were expressed at the mRNA level, resulting in a normal CD1a phenotype at the protein level (Fig E5, *B*, and Fig E4, *A*).

We next isolated whole blood genomic DNA from all family members and sequenced the CD1a gene and 5000bases upstream and downstream using Illumina MiSeq (see Fig E5, *C* [Sanger sequencing] and Table E2 [MiSeq] in this article's Online Repository at www.jacionline.org). Donors 007 and 005 were heterozygous for rs761269454 (Fig E5, *C*, and Table E2), but expressed only the variant form of CD1a (Fig E5, *B*), in contrast to parent 001 and sibling 006, who were also heterozygous but expressed both alleles or at least the normal allele, respectively (Fig E4 and Fig E5, *B*). Intriguingly, we identified a second SNP rs538916791 that introduces a stop codon at amino acid 94 of the CD1a protein. The hereditary distribution of this SNP could explain the CD1a expression pattern: in the presence of the L285P SNP on one allele, the other allele was expressed normally. However, if one allele contained the L285P SNP and the other allele contained the stop codon SNP, as for 005 and 007, only the mutant L285P form could be expressed.

To test whether the L285P mutation was sufficient to abrogate surface CD1a expression, we recombinantly expressed both the reference/wild-type and the mutant forms of CD1a in human embryonic kidney cells (a fibroblast cell line) and K562 cells (a granulocytic/monocytic cell line) (Fig 2, *B*). We chose 2 cell lines to address potential cell-type–specific differences in expression. Flow cytometry analysis showed that only the reference but not the mutant form of CD1a was expressed on the cell surface (Fig 2, *B*), whereas both forms were transcribed equally (Fig E3). Immunohistochemistry of transfected HEK cells confirmed this finding (Fig 2, *C*). Different transfection ratios of normal to mutant CD1a resulted in the expected expression level of normal CD1a and excluded competition at the translational level (Fig 2, *B*).

In summary, we describe complete CD1a deficiency in 2 apparently healthy Vietnamese adults, and have identified a novel mutation responsible for the expression defect. This did not result in any apparent CD1a-related skin abnormalities, or in systemic immune impairment in either individual.

CD1a-restricted T cells specific for the mycobacterial lipopeptide didehydroxymycobactin can be detected in the blood of tuberculin-positive individuals *ex vivo*. Besides a potential role of CD1a-restricted T cells in antibacterial responses, presentation of natural skin lipids to CD1a-autoreactive T cells has been suggested to be essential for maintenance of the skin immune barrier. According to this hypothesis, a skin injury causes CD1a-expressing epidermal LCs to activate dermal CD1a-restricted T cells, resulting in IL-22 secretion, which, in turn, helps to repair any epithelial damage.^6^, ^7^ Moreover, the inflammation caused by bee and wasp venom is mediated via CD1a-restricted self-reactive T cells in the skin. These venoms contain phospholipase A2, which processes skin lipids that are then presented as neoantigens on CD1a, resulting in the activation of CD1a-restricted T cells.^8^

None of the family members described here had a history of tuberculosis, although all are likely to have been exposed because tuberculosis is endemic in the region. Similarly, there was no apparent difference in the occurrence of common skin infections, or in wound healing, between family members displaying different CD1a expression patterns, and no family members recalled unusual reactions to bee or wasp stings.

These findings suggest that it is unlikely that CD1a surface expression is an essential element in the proposed pathway by which LCs are thought to function to maintain the integrity of the skin immune barrier.

## Figures and Tables

**Fig E1:**
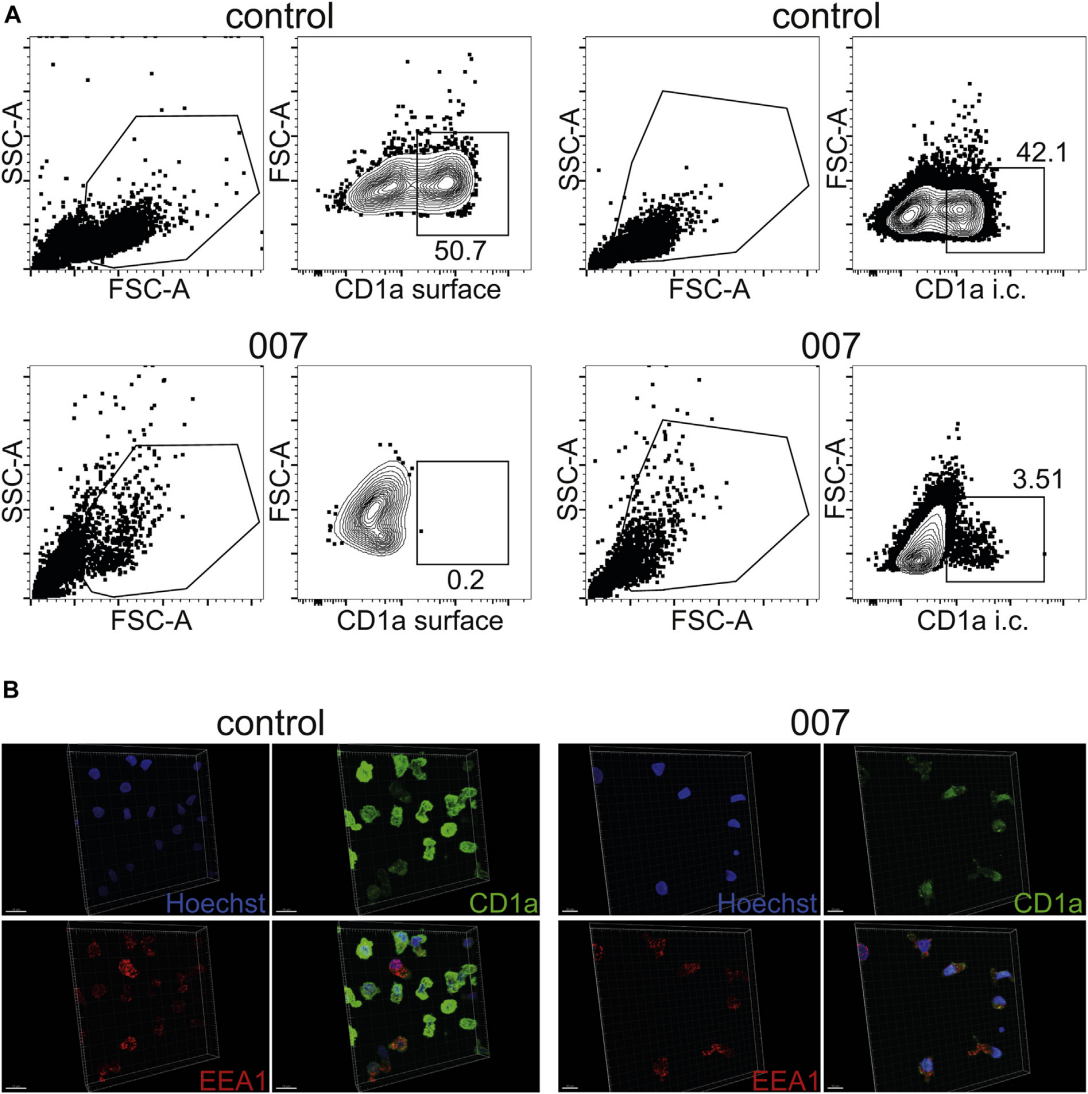
CD1a deficiency is generic and not confined to primary skin APCs. **A,** moDCs from a healthy control donor and from donor 007 were stained either on the surface or intracellularly (i.c.) with anti-CD1a and analyzed by flow cytometry. **B,** Immunohistochemistry of moDCs stained with anti-CD1a *(green)*, anti-EEA, an early endosome marker *(red)*, and Hoechst *(blue)*. Healthy control data are representative of 2 individuals. *EEA*, Early endosome antigen; *FSC-A*, Forward scatter-area; *SSC-A*, side scatter-area.

**Fig E2:**
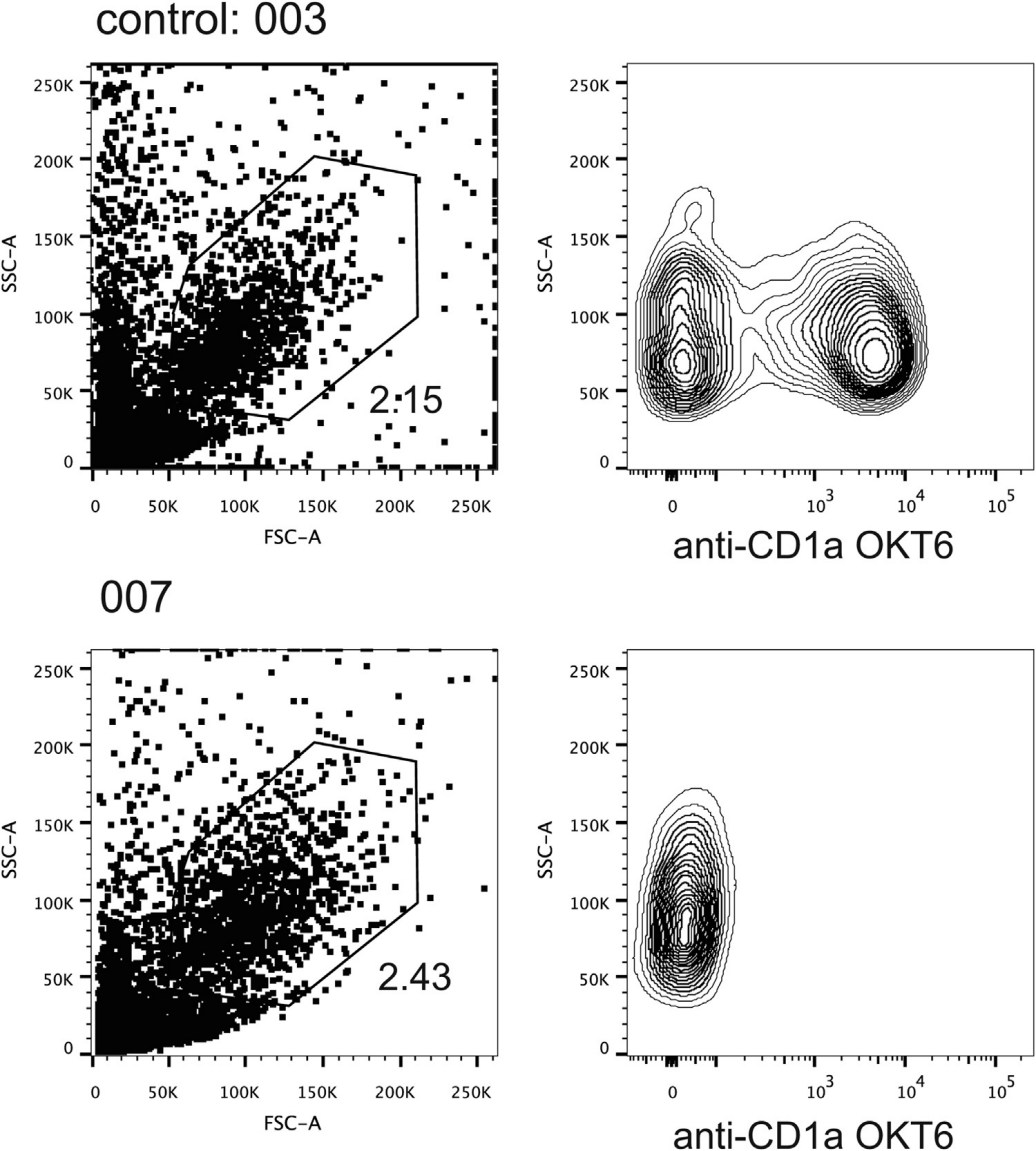
Absence of surface CD1a detection in donor 007 is independent of the antibody clone used. Monocyte-derived DCs from donors 003 and 007 were stained with anti-human CD1a clone OKT6. A positive signal was observed only for donor 003. *FSC-A*, Forward scatter-area; *SSC-A*, side scatter-area.

**Fig E3:**
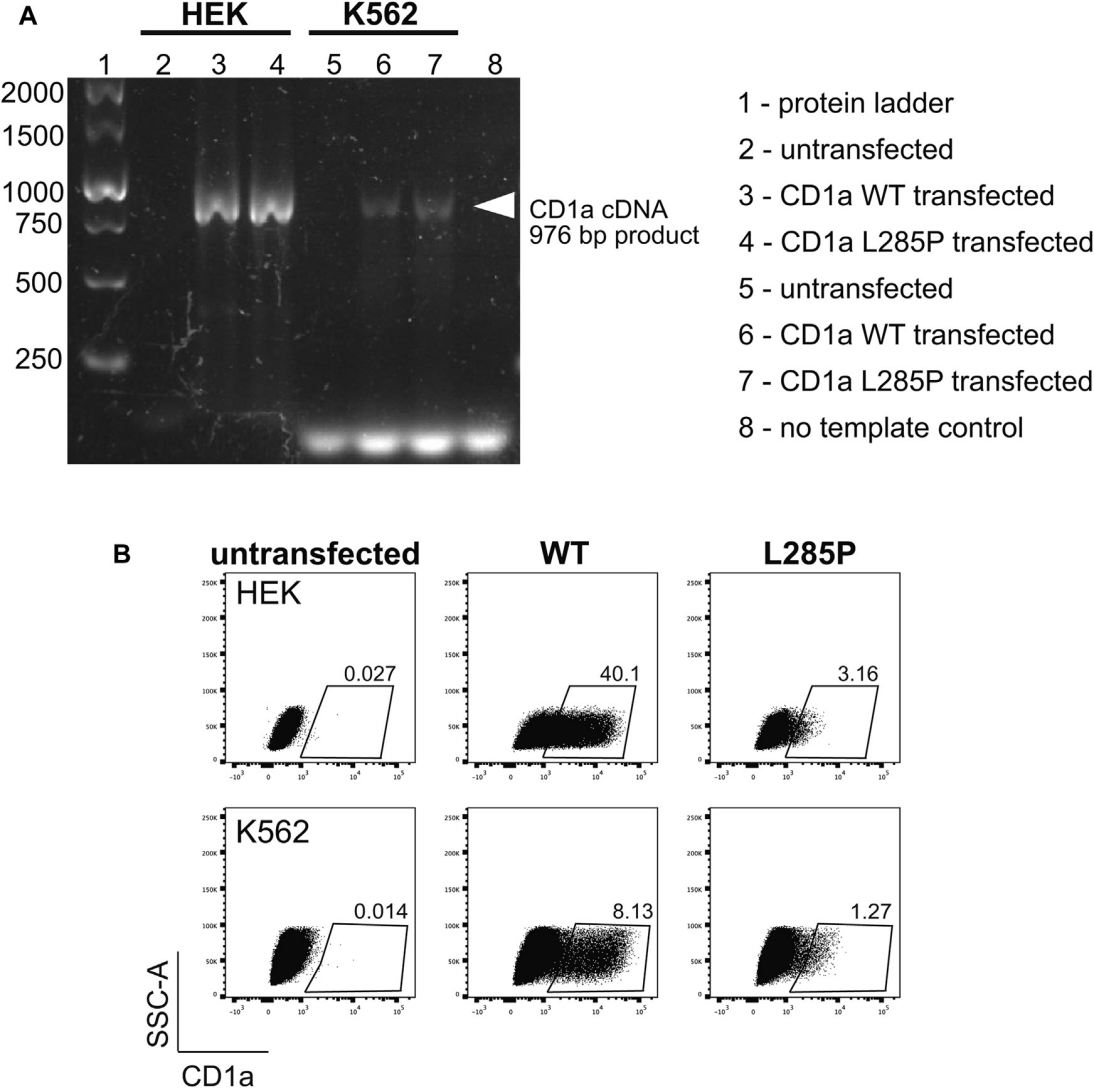
CD1a L285P is transcribed but not expressed as a protein on the cell surface. **A,** mRNA expression of HEK and K562 cells-transfected WT and mutant forms of CD1a. **B,** Detection of WT and mutant CD1a by anti-CD1a clone NA1/34-HLK. HEK and K562 cells were transfected for 72 hours before analyzing the surface expression of CD1a by flow cytometry. The expression pattern is similar to the one detected by anti-CD1a clone HI149 (Fig 2). *HEK*, Human embryonic kidney; *SSC-A*, side scatter-area; *WT*, wild-type.

**Fig E4:**
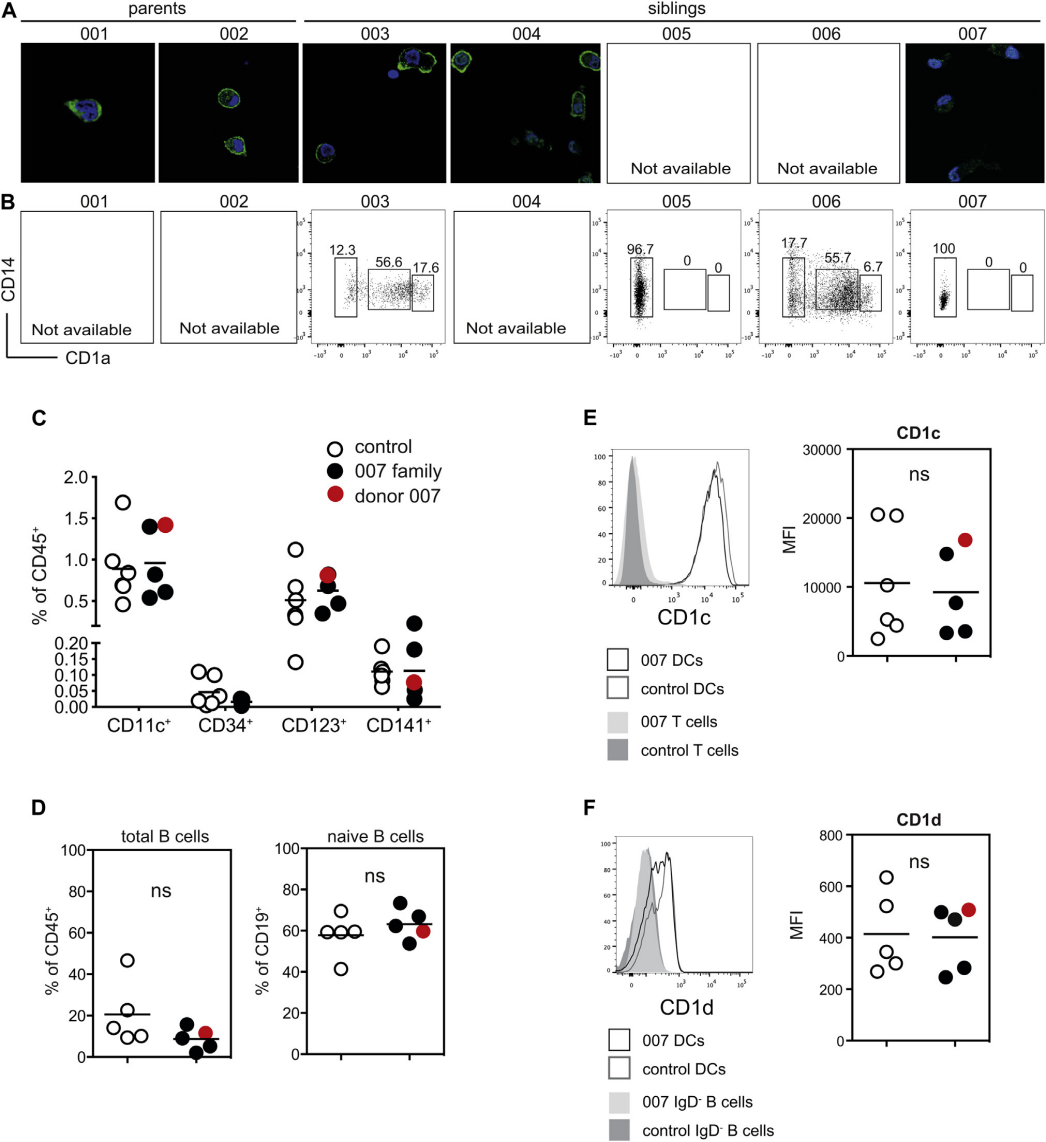
CD1a deficiency is selective and spares CD1c and CD1d expression. CD1a expression on moDCs from affected individuals and family members analyzed by flow cytometry **(A)** and immunofluorescence microscopy **(B)**. **C,** Frequency of peripheral blood myeloid DC subsets, pDCs, and CD34+ stem cells in the affected family (*black* symbols) and healthy controls (open symbols). **D,** Frequency of total B cells and naive B cells (CD19+IgD+) in 007 family members and control donors. CD1c **(E)** and CD1d expression **(F)** on blood DCs from 007 family members and control donors. Representative histograms for 007 and 1 control donor are shown. Donor 007 (with the CD1a defect) is indicated in *red*. Cells from donor 005 were not available for this analysis. *MFI*, Median fluorescence intensity; *ns*, nonsignificant.

**Fig E5:**
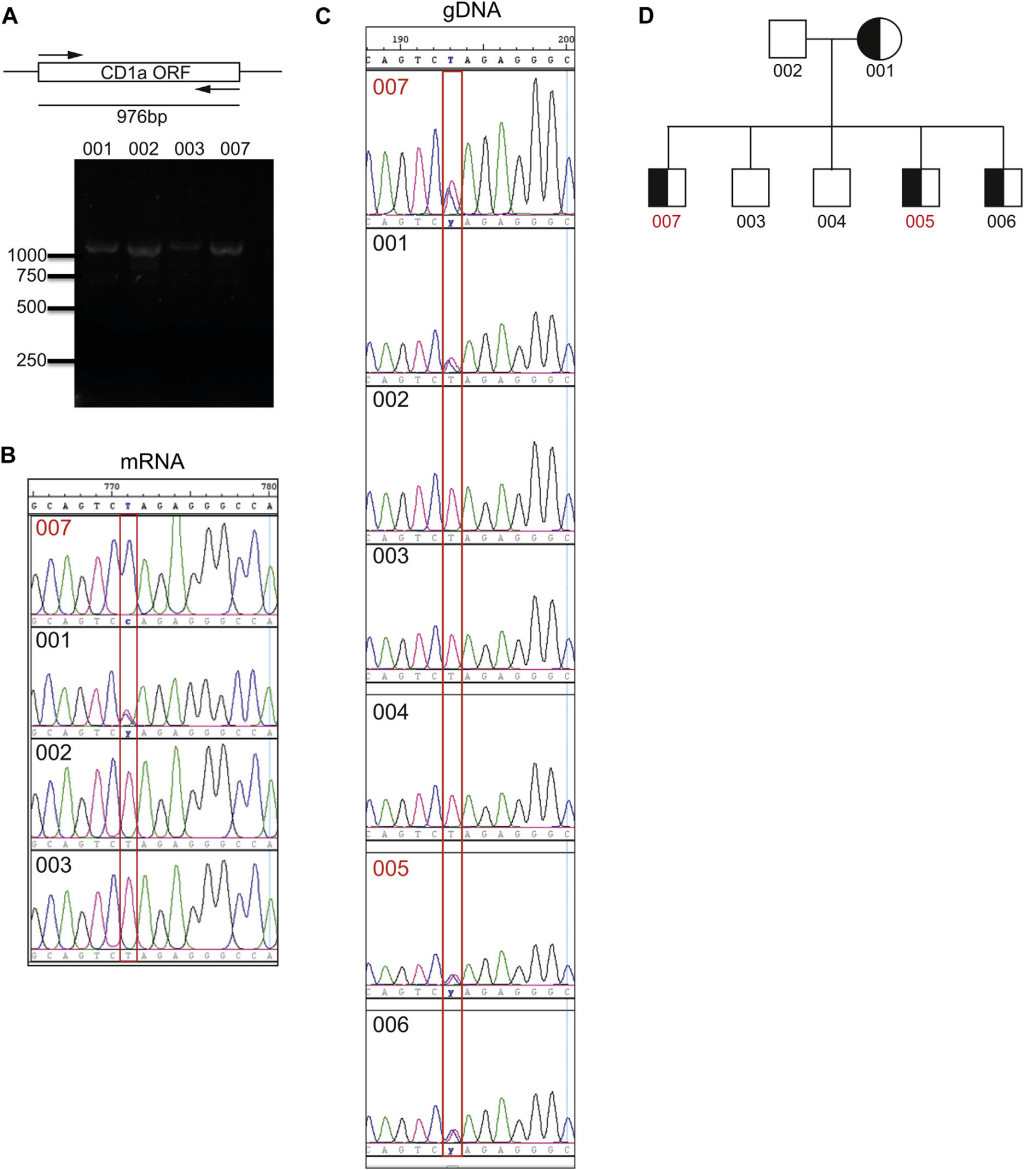
Sequence analysis of CD1a mRNA and gDNA identifies a novel mutation causing CD1a deficiency. **A,** mRNA length of the CD1a ORF amplified from mRNA extracted from moDCs generated from 4 family members. **B,** A thymidine (T) to cytidine (C) mutation identified by mRNA sequencing of donor 007 and family members is indicated with a *red* box. **C,** Sequences of the genomic DNA (gDNA) of family members; the CD1a gene position coding for the mutated mRNA is indicated with a *red* box. **D,** CD1a genotype tree of the family members. The family members with CD1a deficiency are indicated in *red*. *gDNA*, Genomic DNA; *ORF*, Open Reading Frame.

**Table E1:** Summary clinical and laboratory information for the index case (007) and his immediate family

Subject	001	002	003	004	005	006	007—index case∗
CD1a status	rs538916791: C/C rs761269454: T/C Normal CD1a	rs538916791: C/A rs761269454: T/T Normal CD1a	rs538916791: C/A rs761269454: T/T Normal CD1a	rs538916791: C/A rs761269454: T/T Normal CD1a	rs538916791: C/A rs761269454: T/C CD1a deficiency	rs538916791: C/C rs761269454: T/C Normal CD1a	rs538916791: C/A rs761269454: T/C CD1a deficiency
Sex	Female	Male	Male	Male	Male	Male	Male
Year of birth	1959	1956	1991	1982	1986	1985	1989
Occupation	Farmer	Farmer	Businessman	Businessman	Construction worker	Computer repair business	Medical doctor
History (general)	Arthritis, hypertension, vertigo	Parkinson's disease, anal fistula	Unremarkable	Dengue, allergic rhinitis	Gastritis, sinusitis, Bell's palsy	Labyrinthitis	Complex forearm fracture, internal fixation
Skin abscesses or recurrent skin infections?	Occasional, in childhood only	Occasional, in childhood only	No	No	Moderate frequency, in childhood only	Moderate frequency, in childhood only	Moderate frequency, in childhood only
Other skin disorders	Mild acne in the past	Moderate acne in the past	Mild acne (ongoing)	Mild acne and an episode of herpes zoster in the past	Mild acne and folliculitis (ongoing)	Moderate acne (ongoing) Minor fungal skin infections and warts	Mild acne and fungal skin infections in the past
Wound healing	Normal, a few days	Normal, a few days	Normal, a few days	Normal, a few days	Normal, a few days	Normal, a few days	Normal, a few days
Reactions to bee stings	No specific event recalled	No specific event recalled	1-2 events, mild reactions	1-2 events, mild reactions	>10 events, some multiple; mild reactions	>20 events, some multiple; moderate reactions	1-2 events, mild reactions
Reactions to wasp stings	No stings	No stings	No stings	1-2 events, moderate reactions	Once, moderate reaction	1-2 events, moderate reactions	No stings
Examination findings							
General examination	Unremarkable	Parkinsonian	Unremarkable	Unremarkable	Unremarkable	Unremarkable	Unremarkable
BMI	24.6	20.8	20.3	21.5	21.8	19.6	20.3
Visible BCG scar†	No	No	**Yes, normal appearance**	No	No	No	**Yes, normal appearance**
Mantoux (5IU PPD-S)	Not done	Not done	9-mm response	Not done	3-mm response	Not done	10-mm response
Laboratory results							
White blood cells	6.57	9.12	4.76	5.31	5.6	4.47	10.2
Hemoglobin	14	15.8	17.3	16	15.9	16.7	17
Platelets	222	241	161	174	227	127	216
Glucose	4.2	4.4	5.1	5.1	4.8	4.8	5.6
Creatinine	74	85	75	80	94	95	76
AST	31	22	16	18	23	26	20
ALT	21	11	18	28	19	38	40
GGT	8	31	23	23	24	29	26
Cholesterol	4.4	5.1	4.4	4.8	4.2	4.4	4.7
HDL	0.9	1.2	1.3	1.2	**0.8/**0.9	1.3	1.3
LDL	2.8	3.4	2.9	3.4	2.9	2.8	3.2
Triglyceride	1.7	1	1.4	1.5	**2.5/2.6**	1.1	1.3/1.6

Local normal ranges for hematology and biochemistry tests (abnormal results indicated in boldface):

White blood cells: Male/female: 6-10 K/μL.

Hemoglobin: 14.5-15.7 g/dL for men and 13-14 g/dL for women.

Platelets: 201-324 K/μl for men and 211-337 K/μl for women.

Glucose: 3.9-6.4 mmol/L.

Creatinine: 62-120 μmol/L for men and 53-100 μmol/L for women.

AST (aspartate aminotransferase): 0-40 U/L for men and 0-37 U/L for women.

ALT (alanine aminotransferase): 0-40 U/L for men and 0-33 U/L for women.

GGT (gamma glutamyl transferase): 11-50 U/L for men and 7-32 U/L for women.

Cholesterol: 3.9-5.2 mmol/L.

HDL (high density lipoprotein): 0.9-1.78 mmol/L.

LDL (low density lipoprotein): 1.15-3.4 mmol/L.

Triglyceride: 0.46-1.6 mmol/L for men and 0.68-1.88 mmol/L for women.

∗ Additional investigations were performed on the index case (007): electrolytes and plasma protein levels, bone biochemistry, thyroid function tests, immunoglobulin A, M, & G levels, and an abdominal ultrasound were all normal.

† BCG vaccination status uncertain—only subjects 003, 005, and 007 are thought to have received any vaccinations in infancy, but there are no records of what they were given.

**Table E2:** SNP calls for the 13,807 base pairs sequenced in this study

SNP ID	Ref name	Position	snpEff call	Reference base	Alternative base	Donor 001	001_refC	001_altC	001_totC	Donor 002	002_refC	002_altC	002_totC	Donor 003	003_refC	003_altC	003_totC	Donor 004	004_refC	004_altC	004_totC	Donor 005	005_refC	005_altC	005_totC	Donor 006	006_refC	006_altC	006_totC	Donor 007	007_refC	007_altC	007_totC
rs75981383	chr1	158249693	Upstream modifier	C	G					Het	39.20%	40.76%	2495	Het	41.68%	39.89%	3397	Het	41.65%	40.11%	3712	Het	41.14%	37.41%	2144					Het	39.30%	37.89%	3112
rs3181029	chr1	158250175	Upstream modifier	C	T					Het	40.09%	43.90%	4647	Het	42.31%	40.94%	6209	Het	41.97%	42.46%	6562	Het	40.23%	41.98%	3435					Het	38.85%	43.37%	5382
rs3181031	chr1	158250679	Upstream modifier	T	C					Het	42.39%	40.72%	2699													Het	39.08%	42.18%	2257				
rs858998	chr1	158250785	Upstream modifier	C	T	Hom	0.32%	84.07%	5890					Het	41.59%	43.19%	3200	Het	42.90%	41.55%	3427	Het	39.49%	42.36%	1636	Het	41.55%	40.98%	2084	Het	44.08%	38.42%	2829
rs858999	chr1	158251080	Upstream modifier	T	C	Hom	0.10%	81.32%	4165	Hom	0.06%	82.27%	1630	Hom	0.19%	82.93%	2126	Hom	0.05%	83.99%	2130	Hom	0.19%	80.97%	1035	Hom	0.07%	79.49%	1487	Hom	0.09%	82.01%	2196
rs76519430	chr1	158251927	Upstream modifier	G	C					Het	40.53%	39.52%	5428													Het	40.46%	38.47%	5045				
rs79039536	chr1	158252071	Upstream modifier	C	T					Het	44.44%	42.70%	2403													Het	47.66%	41.08%	2157				
rs3136533	chr1	158253270	Upstream modifier	G	A					Het	41.94%	45.31%	1867													Het	41.07%	44.45%	1388				
rs6660005	chr1	158253691	Upstream modifier	A	G					Het	47.37%	43.84%	1104													Het	54.90%	33.48%	938				
rs16840041	chr1	158254269	5′ UTR modifier	G	A					Het	46.42%	41.42%	3141													Het	45.00%	37.76%	2031				
rs366316	chr1	158254492	5′ UTR modifier	A	G					Het	34.42%	45.92%	3190	Het	36.82%	43.47%	4150	Het	37.44%	41.71%	4725	Het	33.22%	42.75%	3064					Het	30.40%	46.16%	2803
rs411089	chr1	158255035	Intron modifier	C	T					Hom	0.49%	85.74%	4052	Het	38.85%	47.87%	4903	Het	40.27%	45.80%	5419	Het	36.44%	48.09%	3271	Het	40.37%	45.65%	2668	Het	34.64%	50.41%	3271
rs2269714	chr1	158255114	Moderate missense: aCc/aTc: T30I	C	T					Het	45.21%	44.37%	3942													Het	43.04%	47.49%	3325				
rs2269715	chr1	158255229	Moderate missense: tgC/tgG: C68W	C	G					Het	42.30%	39.36%	3402													Het	38.29%	38.79%	3643				
**rs538916791**	**chr1**	**158255306**	Stop gained, high nonsense: tCa/tAa: S94*	**C**	**A**					**Het**	**44.79%**	**41.29%**	**1969**	**Het**	**42.17%**	**42.41%**	**2511**	**Het**	**42.91%**	**40.50%**	**2736**	**Het**	**39.91%**	**43.04%**	**1601**					**Het**	**39.86%**	**44.05%**	**1814**
rs2269716	chr1	158255453	Intron modifier	A	T					Het	39.99%	44.55%	2258													Het	42.27%	40.15%	2730				
rs440419	chr1	158255606	Intron modifier	G	T					Hom	0.10%	92.55%	3960	Het	41.70%	47.23%	5461	Het	41.89%	47.31%	5842	Het	41.77%	44.78%	3546	Het	41.03%	45.06%	5062	Het	40.94%	45.41%	4279
rs2269717	chr1	158255730	Intron modifier	T	C					Het	42.37%	47.33%	5863													Het	43.53%	43.87%	7574				
rs11264948	chr1	158256617	Intron modifier	C	A					Het	40.62%	36.45%	4867	Het	38.59%	38.09%	7165	Het	38.79%	36.77%	6992	Het	40.59%	36.08%	3930					Het	38.54%	37.67%	4715
**rs761269454**	**chr1**	**158257035**	**Moderate missense: cTa/cCa: L285P**	**T**	**C**	**Het**	**38.79%**	**42.01%**	**7350**													**Het**	**37.10%**	**42.54%**	**5251**	**Het**	**38.03%**	**42.34%**	**7111**	**Het**	**38.80%**	**40.59%**	**5943**
rs389293	chr1	158258602	Downstream modifier	G	A					Het	44.28%	38.71%	2604	Het	41.72%	41.53%	4293	Het	42.66%	41.76%	4440									Het	39.16%	38.65%	2533
rs550983993	chr1	158259566	Downstream modifier	A	T					Het	45.89%	41.32%	1970													Het	44.19%	41.66%	2331				
rs76706813	chr1	158260035	Downstream modifier	G	A					Het	44.75%	43.31%	4177	Het	44.43%	43.77%	6815	Het	44.31%	42.80%	7260									Het	43.43%	41.75%	4156

Note that ∼20% of the reads were of low quality and not included in the SNP calls after mapping. SNPs in boldface are discussed in the text.

*altC*, Alternative base; *_altC*, percent of total reads with alternative base; *het*, heterogeneous; *homo*, homogeneous; *refC*, reference base; *_refC*, percent of total reads with reference base, *totC,* total number of reads spanning the position.

**Table E3:** Primer sequences for CD1a gene

Genomic coordinates	Target	Forward	Reverse	Amplicon size	Overlap with next amplicon
Chr 1: 158249137-158252175	Amplicon-1	atcaaacctaagctgactcctc	accagacccatctcctctattg	3039	479
Chr 1: 158251697-158255283	Amplicon-2	aagtgttcctgcctttcttccag	taatgtttccagttccttccac	3587	307
Chr 1: 158254976-158257976	Amplicon-3	atggatccccttttctccagattc	ttaatagttgaacatgtggagg	3001	224
Chr 1: 158257752-158261027	Amplicon-4	ggctccagacacacctgaacac	acaggtcaggtattcctaatgtg	3276	488
Chr 1: 158260539-158262943	Amplicon-5	aactgtatccaaagcctgaatg	agtcgtcatttcaggttattgc	2404	NA

*NA*, Not applicable/available.

**Fig 1:**
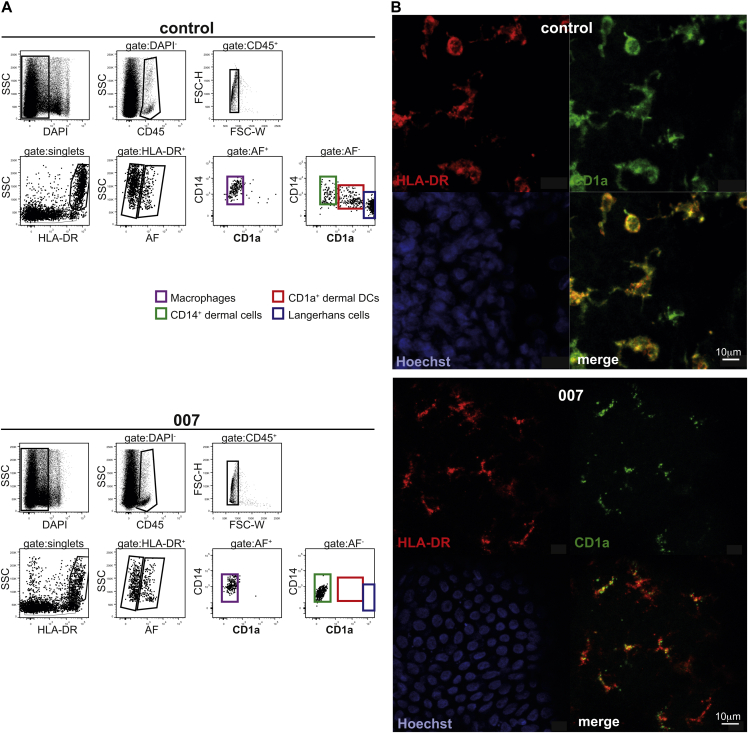
CD1a deficiency on skin DCs of a healthy adult. **A,** Single cells were isolated from skin biopsies from a healthy individual and donor 007 and skin APCs were analyzed by flow cytometry. **B,** Epidermal sheets from a control donor and from donor 007 were stained for HLA-DR *(red)* and CD1a expression *(green)*. Hoechst *(blue)* was used to stain cell nuclei, and samples were analyzed by confocal microscopy. Control data are representative of more than 20 healthy donors. *AF*, Autofluorescence; *DAP*I, 4′-6-diamidino-2-phenylindole, dihydrochloride; *FSC-W*, forward scatter-width; *SSC*, side scatter.

**Fig 2:**
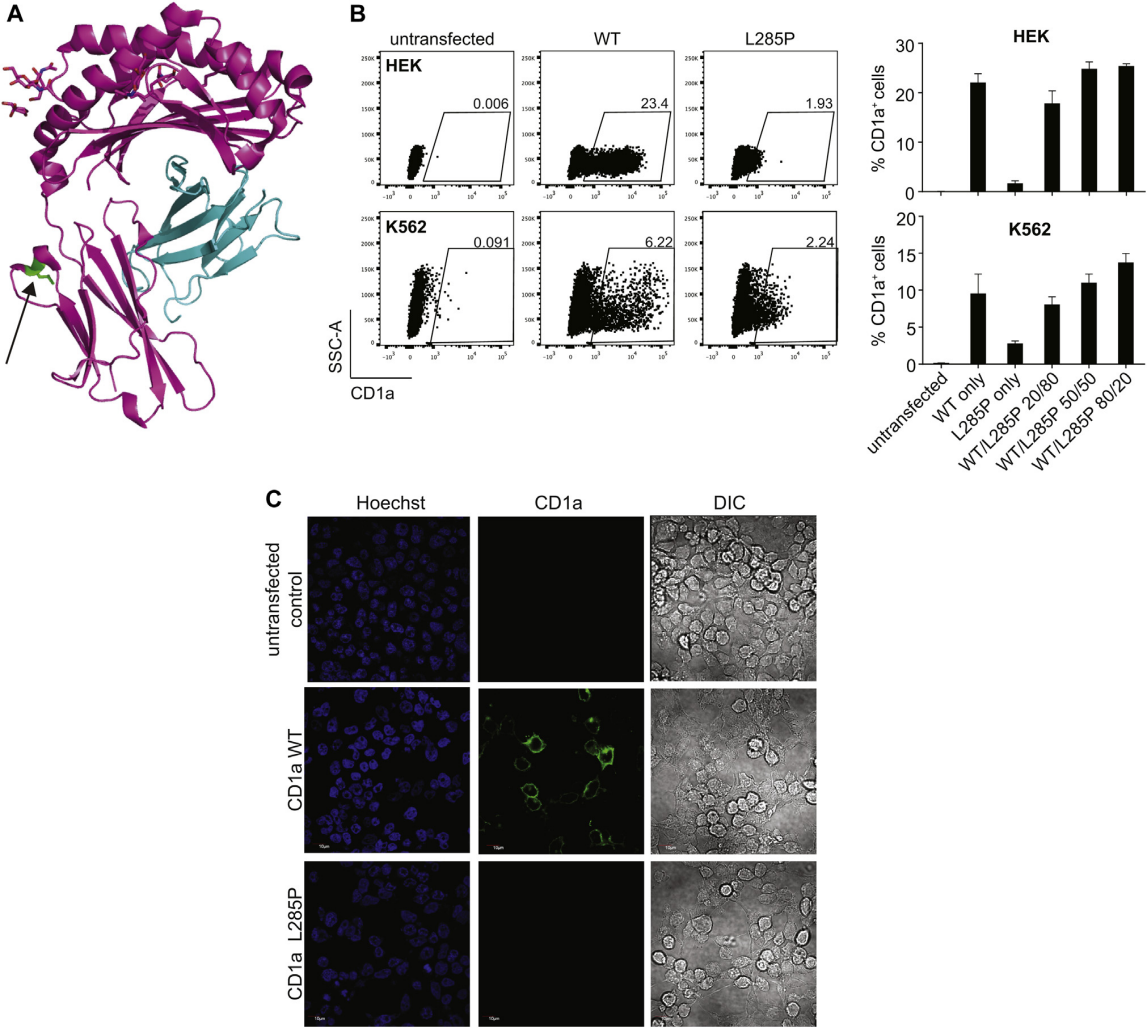
Recombinant expression of the mutant CD1a-L285P reproduces the *in vivo* expression defect. **A,** Structure of the CD1a molecule in complex with a sulfatide (Protein Data Bank 1ONQ). The α domains *(pink)*, β2 microglobulin *(blue)*, and the position of L285P in the α3 subunit *(green)* are shown. **B,** CD1a surface expression of WT and L285P CD1a-transfected HEK and K562 cells analyzed by flow cytometry 24 hours after transfection. Cells were transfected with the indicated ratios of WT and L285P CD1a plasmid. Bar graphs show means ± SEM of % CD1a-expressing cells measured in 2 independent experiments with total n = 4. **C,** CD1a expression on transfected HEK cells analyzed by immunofluorescence microscopy. *HEK*, Human embryonic kidney; *SSC-A*, side scatter-area; *WT*, wild-type.

## Methods

### Clinical methods

To study the index case and his family members, ethical approval was obtained from the Ethical Committee of the Hospital for Tropical Diseases of Ho Chi Minh City and the Oxford Tropical Research Ethics Committee. Following written informed consent, a detailed clinical assessment was performed by a single physician, and a blood sample was obtained for a full hematology/biochemistry panel together with a sample for the immunological studies described below. Four of 7 participants also consented to a shave skin biopsy performed by the same physician.

Control samples consisted of anonymized blood specimens provided by healthy donors to the National University Hospital of Singapore Blood Bank. All donors gave written informed consent.

### Skin biopsies

Shave biopsies were taken under local lignocaine anesthesia using DermaBlades (Personna Medical, AccuTec Blades Inc, Verona, Va). Biopsies were collected in RPMI medium and cut into 2 parts. One part was fixed overnight at 4°C in PBS containing 30% sucrose and 2% paraformaldehyde, then washed in 30% sucrose for 2 hours, and kept in PBS at 4°C until use. The other section of the biopsy was cut into small pieces and digested overnight at 37°C in RPMI medium containing 0.8 mg/mL collagenase (Type IV, Worthington-Biochemical, Lakewood, NJ) and 100 U/mL DNase (Roche, Ho Chi Minh City, Vietnam). Digested tissue was disrupted by manual pipetting, and connective tissue and debris were removed by filtering the cells through a 70-μm filter. Cells were labeled with the following antibodies: HLA-DR-PE-Cy7 (clone L243), CD45-V500 (clone HI30), CD1a-APC (clone HI149), and CD14-PE (clone M5E2) (all from Becton Dickinson, BD Biosciences, San Jose, Calif) before flow cytometry analysis. The gating strategy has been described elsewhere.^E1^

### Differentiation of monocyte-derived DCs, histology, and flow cytometry

PBMCs were isolated by Ficoll density gradient (GE Healthcare Life Science, Singapore) and frozen in liquid nitrogen for later analysis. Monocytes were isolated using CD14-microbead positive selection (STEMCELL Technologies Canada Inc, Singapore). CD14+ monocytes were cultured in RPMI medium supplemented with 10% FCS with 50 ng/mL recombinant human GM-CSF and 10 ng/mL IL-4 (both from Immuno Tools, Friesoythe, Germany) for 6 to 7 days to generate moDCs. Anti-CD1a clone HI149 (Biomarkers and Immunoassays, Biolegend, San Diego, Calif) or clone OKT6^E2^ was used for flow cytometry and histology.

For microscopy analysis, cells were seeded on Poly-l-Lysine–coated chamber slides (Ibidi, Planegg/Martinsried, Germany) and fixed with 4% paraformaldehyde for 20 minutes. Cells were then permeabilized with 0.1% Triton X-100 and blocked with 3% BSA for 2 hours before the antibody was added at room temperature and incubated for 2 hours. Hoechst (200 ng/mL; Invitrogen, Thermo Fisher Scientific, Carlsbad, Calif) was added for 5 minutes and slides were washed 3 times before addition of Prolong Gold (Life Technologies Corporation, Thermo Fisher Scientific, Carlsbad, Calif). Images were taken on an Olympus IX81 confocal microscope.

### PBMC flow cytometry analysis

PBMCs were isolated using Ficoll (GE Healthcare) or cell preparation tubes (Becton Dickinson) and frozen for later analysis. Thawed cells were stained and analyzed using a FACS-LSRII (Becton Dickinson). For the DC subset analysis, the negative fractions of the CD14-positive sort (see moDC generation) were used. DC subsets were determined following a previously described gating strategy^E3^ that involved using antibodies binding to lineage (CD19 [clone HIB-19], CD20 [clone 2H7], CD3 [clone UCHT1], CD56 [clone MEM188]), CD45 (clone HI30), HLA-DR (clone L243), CD14 (clone RM052; Life Sciences and Diagnostics, Beckman Coulter, Brea, Calif), CD11c (clone B-ly6), CD1c (clone L161), CD141 (clone AD5-14H12; MACS FlowCytometry, Miltenyi Biotec, Singapore), CD34 (clone 563; BD Pharmingen, BD Biosciences, San Jose, Calif), CD123 (clone 7G3), and Hoechst. For the analysis of B cells and the analysis of CD1d expression, PBMCs were stained with CD45 (clone HI30), CD19 (clone HIB19; BD Pharmingen), CD1c (clone L161), CD1d (clone 51.1), CD3 (clone UCHT1), CD14 (clone RM052; Beckman Coulter), and Hoechst. All antibodies were purchased from Biolegend, unless otherwise stated.

### Sequencing of messenger RNA and genomic DNA

For mRNA sequencing, moDCs were collected into catch buffer (10 mM Tris, pH 8, RNase inhibitor RNasin [Promega Corporation, Madison, Wis]) and frozen at −80°C until use. cDNA was generated and amplified using the One-Step PCR Kit (Qiagen, Hilden, Germany) with the following primers: 5′-CTA CTT CCA TTG TTA GCT GTT CTC CC and 5′-TGT CTT AAC AGA AAC AGC GTT TCC T. The PCR product was loaded on an agarose gel and the product was isolated with a gel extraction kit (Qiagen).

Genomic DNA was isolated from whole blood using the DNeasy Blood & Tissue Kit (Qiagen). Sequencing of the CD1a gene was first performed using overlapping primers and sequencing the PCR products with Sanger sequencing (Fig E5, *C*).

For a deeper coverage, we used Illumina MiSeq to sequence the CD1a gene and approximately 5-kb region upstream and downstream of the coding regions, inclusive of the 5′ and 3′ UTRs. The entire 13.8-kb genomic sequences (->hg38_refGene_NM_001763 range=chr1:158249137-158262944) were subdivided into 5 shorter regions and were amplified for library preparation. Primer sequences and their positions can be found in the table below (Table E3).

Amplification was performed using 10 ng of total genomic DNA prepared from blood as template using LongAmp Taq DNA polymerase (New England Biolabs, Singapore) according to manufacturer's instructions. PCR cycling conditions for amplicon regions 1, 3, 4, and 5 were 94°C for 30 seconds (initial denaturation), followed by 25 cycles of 94°C for 15 seconds (denaturation), 58°C for 30 seconds (annealing), and 65°C for 30 seconds (extension), with a final elongation of 65°C for 10 minutes. For amplifying the amplicon 2 region, the cycling profile was similar to above except that the annealing was carried out at a higher temperature (60°C for 30 seconds). Amplified products were purified using Agencourt Ampure XP beads (Beckman Coulter). Equimolar amounts of PCR products were pooled together for each sample separately for library preparation.

Libraries of pooled amplicons for each sample were prepared using the Nextera XT kit (Illumina, San Diego, Calif) according to manufacturer's instructions. Libraries were constructed using 0.8 ng of pooled amplicons as starting material. Briefly, fragmentation of template DNA (5 μL) was carried out in 10 μL of Tagment DNA buffer using 5 μL of Amplicon Tagment Mix. Indexes were added to Tagmented DNA using 12 PCR cycles. The amplified and indexed libraries were purified and size selected using Agencourt Ampure XP beads (Beckman Coulter). The length distribution of the libraries was monitored using DNA 1000 kits on the Agilent 2100 Bioanalyzer (Agilent Technologies, Singapore). Equimolar amounts of purified libraries were pooled and sequenced using indexed PE sequencing runs of 2 × 250bp on an Illumina MiSeq Personal Sequencer (MiSeq Control Software Version 2.4.1.3). Each individual sample was sequenced at an average depth of 2.3 million reads to detect SNPs in the genomic region of interest.

### Data analysis

MiSeq reads were mapped to the HG38 reference genome with bowtie 2.^E4^ SNP calls were made with Samtools Mpileup and BCFtools.^E5^ Annotation of rs numbers was performed with an in-house custom script and data from dbSNP for the targeted region. SNP function was determined with snpEff and the Gencode V24 annotation.^E6^ The data from this study are available in the NCBI BioProject database (ID: 315777).

### Database screen for L285P prevalence

A comprehensive genetic database comprising more than 2000 Vietnamese individuals genotyped with the Illumina Human Exome arrayE7, E8 was screened but did not reveal the CD1a 285L location to be polymorphic. Further examination of exome-sequenced East Asian samples,^E9^ as well as the 1000 Genomes project Phase 3 cosmopolitan database,^E10^ also did not reveal any polymorphisms in the CD1 285L genomic location.

### Generation of L285P mutant and expression in HEK and K562 cells

Construction of a BCMGSneo CD1a-expressing plasmid has been described elsewhere.^E11^ To introduce the L285P mutant, an overlap PCR was performed using primer pair CD1a_XhoI_FOR 5′-CTTCTCGAGATGCTGTTTTTGCTACTTCC-3′ and CD1a_mut_REV 5′-ATGTCCTGGCCCTCTGGACTGCTGTGCTTCAC-3′ and primer pair CD1a_NotI_REV 5′-CCACAGCGGCCGCTTAACAGAAACAGCGTTTC-3′ and CD1a_mut_FOR 5′-TGAAGCACAGCAGTCCAGAGGGCCAGGACATC-3′. Products were purified, mixed, and used as a template for an overlap PCR using primer pair CD1a_XhoI_FOR and CD1a_NotI_REV. The final product was digested with XhoI and NotI and subsequently ligated into the parental vector.

## References

[1] De Libero G., Mori L. (2012). Novel insights into lipid antigen presentation. Trends Immunol.

[2] Romani N., Brunner P.M., Stingl G. (2012). Changing views of the role of Langerhans cells. J Investig Dermatol.

[3] Seneschal J., Clark R.A., Gehad A., Baecher-Allan C.M., Kupper T.S. (2012). Human epidermal Langerhans cells maintain immune homeostasis in skin by activating skin resident regulatory T cells. Immunity.

[4] Haniffa M., Collin M., Ginhoux F. (2013). Ontogeny and functional specialization of dendritic cells in human and mouse. Adv Immunol.

[5] Klechevsky E., Morita R., Liu M., Cao Y., Coquery S., Thompson-Snipes L. (2008). Functional specializations of human epidermal Langerhans cells and CD14+ dermal dendritic cells. Immunity.

[6] de Jong A., Cheng T.Y., Huang S., Gras S., Birkinshaw R.W., Kasmar A.G. (2014). CD1a-autoreactive T cells recognize natural skin oils that function as headless antigens. Nat Immunol.

[7] de Jong A., Pena-Cruz V., Cheng T.Y., Clark R.A., Van Rhijn I., Moody D.B. (2010). CD1a-autoreactive T cells are a normal component of the human alphabeta T cell repertoire. Nat Immunol.

[8] Bourgeois E.A., Subramaniam S., Cheng T.Y., De Jong A., Layre E., Ly D. (2015). Bee venom processes human skin lipids for presentation by CD1a. J Exp Med.

